# Trans-Anastomotic Drainage Tube Placement After Hand-Sewn Anastomosis in Patients Undergoing Intersphincteric Resection for Low Rectal Cancer: An Alternative Drainage Method

**DOI:** 10.3389/fonc.2022.872120

**Published:** 2022-07-28

**Authors:** Xinjian Zhong, Xiaoyu Xie, Hang Hu, Yi Li, Shunhua Tian, Qun Qian, Congqing Jiang, Xianghai Ren

**Affiliations:** ^1^ Department of Colorectal and Anal Surgery, Zhongnan Hospital of Wuhan University, Wuhan, China; ^2^ Clinical Center of Intestinal and Colorectal Diseases of Hubei Province, Wuhan, China; ^3^ Key Laboratory of Intestinal and Colorectal Diseases of Hubei Province, Wuhan, China; ^4^ Colorectal and Anal Disease Research Center, Medical School of Wuhan University, Wuhan, China; ^5^ Quality Control Center of Colorectal and Anal Surgery, Health Commission of Hubei Province, Wuhan, China

**Keywords:** intersphincteric resection, anastomotic leakage, trans-anastomotic drainage tube, anal function, complication

## Abstract

Anastomotic leakage (AL) is a common complication after intersphincteric resection (ISR). It significantly reduces quality of life and causes great distress to patients. Although traditional drainage (e.g., anal and pelvic catheters) may reduce the impact of AL to some extent, their role in reducing the incidence of AL remains controversial. In this study, we developed a novel drainage technique involving the placement of drainage tubes through the gap between sutures during handsewn anastomosis, to reduce the occurrence of anastomotic leakage. We retrospectively analyzed 34 consecutive patients who underwent intersphincteric resection requiring handsewn anastomosis between February 1, 2017, and January 1, 2021. Patients were classified into the trans-anastomotic drainage tube group (TADT, n = 14) and the non-TADT group (n = 20) based on whether trans-anastomotic tube placement was performed. The incidence of postoperative complications, such as AL, was compared between the two groups, and anal function of patients at 1-year post-ISR was evaluated. Six cases of AL occurred in the non-TADT group, while none occurred in the TADT group; this difference was statistically significant *(p*=0.031). The TADT group also had a shorter hospital stay (*p*=0.007). There were no other significant intergroup differences in operation time, blood loss, pain score, anastomotic stenosis, intestinal obstruction, or incidence of wound infection. In the 30 patients (88.2%) evaluated for anal function, there were no significant intergroup differences in stool frequency, urgency, daytime/nocturnal soiling, Wexner incontinence score, or Kirwan grading. Taken together, trans-anastomotic tube placement is a novel drainage method that may reduce AL after ISR requiring handsewn anastomosis and without adversely affecting anal function.

## Introduction

In 1994, Schiessel (1) first reported the use of intersphincteric resection (ISR) for the treatment of low rectal cancer, as an extreme anus-preserving procedure. In this surgery, dissection and resection are performed between the internal and external anal sphincters to obtain a large tumor margin distance. This helps achieve anal preservation in patients with low rectal cancer, which was not feasible earlier with conventional anterior resection (2). However, postoperative anastomotic leakage (AL) is one of the common and severe complications of ISR (3). The reported incidence of AL after ISR is variable, ranging from 0.9% to 48% (4–7). Not only can AL potentially cause severe life-threatening abdominal and pelvic infections, it is also associated with the risk of chronic anal function impairment. Therefore, AL significantly reduces quality of life and causes great distress to patients.

Surgeons have repeatedly attempted to reduce the incidence of postoperative AL for rectal cancer. The placement of a drainage catheter (e.g., anal and pelvic catheters) may be an important, simple, and feasible method to reduce the incidence of AL. A meta-analysis published in 2019 pooled the clinical data of 10,867 patients with rectal cancer and suggested that transanal drainage tubeplacement after rectal cancer surgery could effectively reduce the incidence of AL (8). However, a prospective study in 2020 and a randomized controlled trial in 2021 both published opposing views regarding transanal drainage tube placement as a protective factor against AL (9, 10).

Pelvic drainage is another conventional drainage method that can reduce the incidence of pelvic hematoma and infections, alleviate clinical symptoms of AL, and contribute to the treatment of AL. However, most clinical studies or meta-analyses indicate that conventional pelvic drainage does not reduce the incidence of AL (11–13). After ISR, a narrow gap is formed between the intestinal canal and external sphincter below the level of the levator ani hiatus. This makes it difficult for conventional pelvic catheters to drain adequately and increases the risk of fluid accumulation and infections, thereby increasing the risk of AL. To address this issue, we modified the pelvic drainage procedure for patients undergoing ISR with handsewn anastomosis, by placing 2–4 small drainage tubes from bottom to top (until the tubes reached the plane of the pelvic levator ani hiatus) while placing interrupted sutures on the reconstructed intestinal tract, in the hopes of achieving good and adequate drainage.

This study aims to preliminarily explore whether this novel drainage method can effectively reduce the incidence of AL after ISR surgery, and to evaluate the safety and feasibility of this method.

## Materials and Methods

We retrospectively analyzed the clinical data of consecutive patients with low rectal cancer who underwent ISR at our center from February 1, 2017, to January 1, 2021. Inclusion criteria were as follows: 1) age above 18 years; 2) diagnosis of low rectal cancer type II or type III according to the Rullier classification system (14) and history of ISR surgery; 3) digestive tract reconstruction completed with coloanal handsewn anastomosis; and 4) provision of consent by signing the informed consent form. Exclusion criteria were as follows: 1) ISR with stapled coloanal anastomosis; 2) presence of significant preoperative anal function impairment; 3) presence of severe mental disorders or communication barriers and unable to complete follow-up evaluations accurately; and 4) patients lost to follow-up or whose medical records were incomplete, thus affecting analysis.

All patients underwent standardized preoperative evaluation, including physical examination, biopsy, endorectal ultrasonography, rectal magnetic resonance imaging (MRI), and thoracic and abdominal computed tomography (CT). Patients who underwent neoadjuvant chemoradiotherapy rested for 8–12 weeks before the elective ISR surgery. None of the patients received long-term oral glucocorticoids, non-steroidal anti-inflammatory drugs, or targeted therapy. Polyethylene glycol was administered orally for bowel preparation and antibiotics were administered 30 min to 1 h before surgery to prevent infections. This study was approved by the Ethics Committee of Zhongnan Hospital of Wuhan University. All patients signed the informed consent form.

### Surgical Technique

The surgery was performed by a fixed team. Our team has had experience more than 150 cases of laparoscopic low rectal sphincter-preserving surgery (tumor distance from anal verge < 5cm). The surgical steps of ISR have been described in detail in previous studies (15). Briefly, the steps of ISR include laparoscopic total mesorectal excision, ISR, manual intestinal reconstruction, and ileostomy. After pelvic dissection is performed using the laparoscopic abdominal approach, the internal sphincter is divided transanally, 1–2 cm distally from the tumor. Under direct vision, intersphincteric dissection is performed upward until transabdominal surgical dissection plane is reached. The tumor specimen is then removed from the anal canal and coloanal anastomosis is performed manually with intermittent sutures, using a 2-0 absorbable suture.

**Figure 1:**
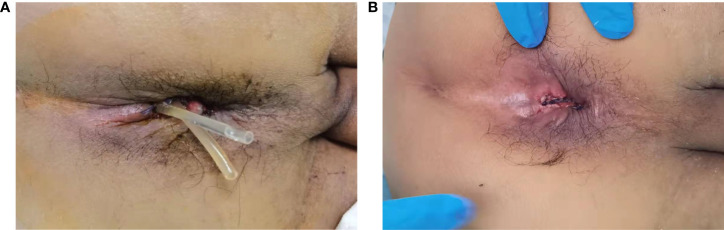
**(A)**, External appearance of the anus after the placement of trans-anastomotic drainage tube postoperative day 1. **(B)**, External appearance of the anus after removing the trans-anastomotic drainage tube.

In the trans-anastomotic drainage tubes (TADT) group, during the intermittent sutures, we placed 2-4 additional infusion tubes (polyvinylamine-chloride tube approximately 10 cm in length and 4 mm in diameter) with multiple-side-hole at the 2 o’clock, 5 o’clock, 7 o’clock, and 10 o’clock positions of anastomosis. The drainage tubes were fixed during suturing to prevent displacement. These tubes passed through the suture gap until they reached the plane of the pelvic levator ani hiatus (**Figure 1A**). Patients without trans-anastomotic drainage tubes placement were assigned to the non-TADT group. All patients underwent prophylactic ileostomy and routine placement of transabdominal pelvic drainage tubes.

### Postoperative Course

**Figure 2:**
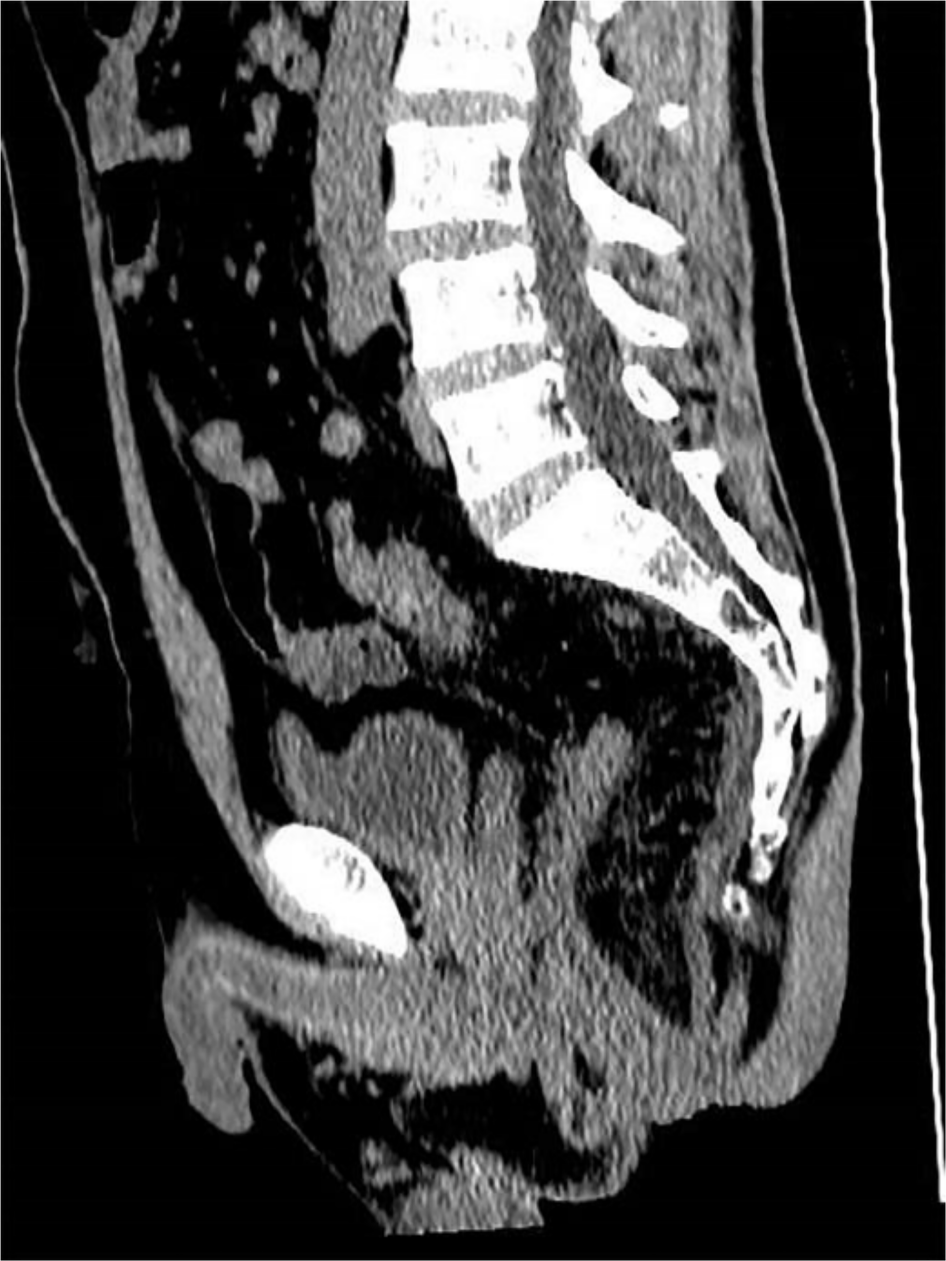
CT image after removing the trans-anastomotic drainage tube (postoperatively day 7).

Postoperatively, all patients were routinely administered antibiotics and total parenteral nutrition, while patients with hypoproteinemia were administered an albumin infusion. After surgery, close attention was paid to the color and volume of the drainage fluid from the pelvic drain in both groups. In addition, for patients in the TADT group, we also observed the drainage status of the anastomotic drainage tubes; in particular, we were watchful for tube dislocation, invagination, or folding. TADTs were flushed with normal saline 20ml once or twice a day for 5–7 days postoperatively to maintain cleanliness of the area around the anastomosis, ensure adequate drainage, and prevent blockages secondary to deposition of foreign bodies. If the anastomosis healed well, the drainage tubes were removed on postoperative days 5–7, depending on the status of the patient (**Figure 1B**). Digital rectal examination (DRE), pelvic CT, or B-scan ultrasonography was routinely performed prior to discharge to assess anastomotic healing and determine development of any complications (**Figure 2**). The standard of good healing of the anastomosis is that the intestinal wall at the anastomosis between colon and rectum/anus is integrity, and there is no pelvic abscess around the anastomosis (16).

**Figure 3:**
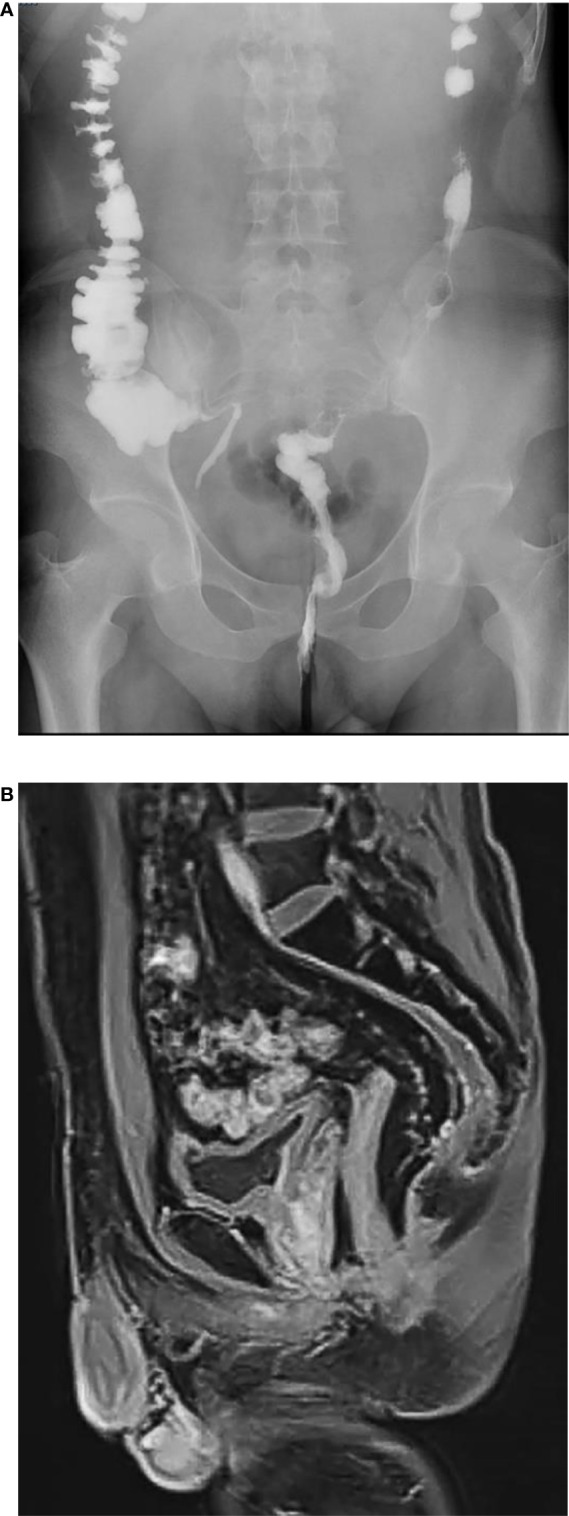
**(A)**, X-ray iodized water radiography of digestive tract showed no delayed anastomotic leakage in the trans-anastomotic drainage tube (TADT) group (six months after operation). **(B)**, MRI showed no obviously delayed anastomotic leakage in the trans-anastomotic drainage tube (TADT) group six months after operation.

During tumor reexamination in the 3-6 months postoperatively, the entire large intestine, including the anastomosis, was reevaluated (**Figure 3**). If no significant abnormalities were detected, ileostomy closure was performed.

### Follow-Up and Functional Assessment

Follow-up visits were conducted every 3 months for the first 2 years, every 6 months for the following 3 years, and annually thereafter. Physical examination (DRE), pelvic MRI, and related hematological investigations were performed in all patients. Anorectal function was also evaluated. After the ileostomy closure surgery, colonoscopy was performed annually if no significant complications occurred.

### Statistical Analyses

All statistical analyses were performed using IBM SPSS version 21.0 (IBM Corp., Armonk, NY, USA). The Fisher’s exact test was used to compare variables expressed as proportions. The Mann–Whitney U test and the Student’s t-test were used to compare nonparametric and parametric variables between the two groups, respectively. All p-values were derived from two-tailed analyses, with statistical significance accepted at p < 0.05.

## Results

### Patient Characteristics

A total of 34 consecutive patients were included in this study, with 14 cases in the TADT group and 20 in the non-TADT group. There were no significant differences between TADT and non-TADT groups with respect to age (56.9 ± 7.0 vs. 61.4 ± 9.0 years old; *p* = 0.128), sex (male/female: 8/6 vs. 10/10; *p* = 0.738), American Society of Anesthesiology (ASA) score (*p* = 0.861), body mass index (BMI) (21.0 ± 2.8 vs. 22.1 ± 2.3; *p* = 0.238), preoperative laboratory counts (preoperative serum CEA/CA199), distance from the lower edge of the tumor to the anal margin (3.5 ± 0.4 vs. 3.5 ± 0.4 cm; *p* = 0.941), tumor size (3.1 ± 0.5 vs. 3.4 ± 0.7 cm; *p* = 0.211), TNM staging (*p* = 0.874), or history of neoadjuvant therapy prior to surgery (21.4% vs. 20.0%; *p* = 1.000) (**Table 1**).

**Table 1:** Patient characteristics (n = 34).

characteristic	non-TADT group (*n* = 20)	TADT group (*n *= 14)	*p* value
Age (year)	61.4 ± 9.0	56.9 ± 7.0	0.128
Gender, *n* (%)			0.738
Male	10 (50)	8 (57.1)	
Female	10 (50)	6 (42.9)	
ASA score, *n* (%)			0.861
I	13 (65)	8 (57.1)	
II	6 (30)	5 (35.7)	
III	1 (5)	1 (7.1)	
Body mass index (kg/m2)	22.1 ± 2.3	21.0 ± 2.8	0.238
Height (cm)	166.3 ± 6.6	163.0 ± 8.1	0.199
Weight (kg)	61.2 ± 8.1	56.2 ± 10.9	0.136
Preoperative serum CEA (ng/ml)	3.5 ± 2.8	4.3 ± 4.5	0.54
Preoperative serum CA199 (IU/ml)	9.9 ± 8.2	6.4 ± 3.7	0.143
Diabetes, *n* (%)	2 (10)	1 (7.1)	1
Hypertension, *n* (%)	5 (25)	3 (21.4)	1
Smoking, *n* (%)	2 (10)	2 (14.3)	1
Hemoglobin level (g/L)	132.5 ± 14.9	126.3 ± 15.7	0.252
Albumin levels (g/L)	41.3 ± 2.9	41.1 ± 2.6	0.82
Distance from anal verge (cm)	3.5 ± 0.4	3.5 ± 0.4	0.941
Preoperative chemoradiotherapy, *n* (%)	4 (20)	3 (21.4)	1
Tumor size (cm)	3.4 ± 0.7	3.1 ± 0.5	0.211
Mean Wexner incontinence score (preoperative)	0.3 ± 0.6	0.2 ± 0.4	0.84
Blood transfusion	1 (5)	1 (7.1)	1
pN stage, *n* (%)			0.627
pN_0_	17 (85)	13 (92.9)	
pN+	3 (15)	1 (7.1)	
pT stage, *n* (%)			1
PCR*	1 (5)	0	
T1	1 (5)	0	
T2	10 (50)	8 (57.1)	
T3	8 (40)	6 (42.9)	
pTNM stage, *n* (%)			0.874
PCR*	1 (5)	0	
I	10 (50)	8 (57.1)	
II	6 (30)	5 (35.7)	
III	3 (15)	1 (7.1)	

ASA, American Society of Anesthesiologists.

Values are means ± standard deviations or medians with ranges in parentheses.

* PCR, pathological complete remission.

### Operative and Postoperative Outcomes

**Table 2** shows the intraoperative status and postoperative complications. There was no significant difference in the operative time (256 ± 11 vs. 249 ± 16 min; *p* =0.192) or intraoperative blood loss (62.5 vs. 40 ml; *p* =0.192) between the TADT and non-TADT groups. In terms of postoperative complications, there were no significant differences between the two groups in the incidence of surgical site infections (7.1% vs. 5.0%; *p*=1.000), intestinal obstruction (13.8 ± 4.4 vs. 7.1% vs. 5.0%; *p*=1.000), or anastomotic stricture (0 vs. 20%; *p*=0.126). However, the incidence of postoperative AL was significantly increased (30% vs. 0%, *p*=0.031) and postoperative hospital stay was significantly prolonged (13.8 ± 4.4 vs. 10.6 ± 1.7 days; *p*=0.007) in the non-TADT group than in the TADT group. According to the grade of AL, all the 6 patients were grade B AL (16). After upgrading antibiotics, strengthening nutrition, reflux pelvic lavage and other conservative treatments, all the patients were cured before discharge.

**Table 2:** Short-term results (n = 34).

characteristic	non-TADT group (*n* = 20)	TADT group (*n* = 14)	*p* value
Operative time (min)	249 ± 16	256 ± 11	0.135
Blood loss (ml)	40 (20–300)	62.5 (25–280)	0.192
Wound infection	1 (5)	1 (7.1)	1
Ileus	1 (5)	1 (7.1)	1
Anastomotic leakage	6 (30)	0	**0.031**
Postoperative stay (day)	13.8 ± 4.4	10.6 ± 1.7	**0.007**
Anastomotic stricture	4 (20)	0	0.126
Pain score			1
1–3	17	12	
4–6	3	2	
7–10	0	0	

Values are means ± standard deviations or medians with ranges in parentheses

Bold value: p < 0.05.

### Anal Function

The function of the reconstructed anus may gradually improve over time after surgery. To reduce the impact of differences in follow-up durations on the anal function between the two groups, we compared the anal function of patients who had undergone ileostomy closure, one year after ISR. A total of 30 patients (88.2%) met the above criteria and were analyzed; four patients were not included in the comparison since they underwent ISR less than one year prior. Postoperative anal function is shown in **Table 3**, the baseline characteristics between subgroups were comparable (the detailed baseline characteristics were shown in **Supplementary Table S1**). Although the TADT group outperformed the non-TADT group in anal function evaluation on stool frequency (*p*=0.949), urgency (16.7% vs. 22.2%; *p*=1.000), and Wexner incontinence score (6.8 ± 2.7 vs. 7.4 ± 2.7; *p*=0.581), the differences were not statistically significant. The proportions of Grade 2 and Grade 3 cases as per the Kirwan classification were relatively high in both groups, and the difference was not statistically significant (*p*>0.05). No patient required colostomy due to severe fecal incontinence (Grade 5). Since AL may affect anal function and confound the effects of anastomotic tube placement on anal function, we analyzed the influence of TADT and non-TADT on anal function in patients without AL. Twelve patients without AL in each group of TADT and non-TADT were compared. **Table 4** depicts the postoperative anal function in the two groups of patients without AL; the baseline characteristics were comparable (the detailed baseline characteristics were shown in **Supplementary Table S2**), and no statistically significant differences with respect to postoperative anal function such as Kirwan grade (*p*=0.572) and Wexner incontinence score (6.8 ± 2.7 vs. 6.0 ± 2.0; *p*=0.395), frequency (*p*=1.000), anti-diarrhea medication (8.3% vs. 0; *p*=1.000), urgency (16.7% vs. 0; *p*=0.478) and soiling were found between the TADT and non-TADT subgroups.

**Table 3:** Anal functional outcomes (n = 30).

Characteristic	non-TADT group (*n* = 18)	TADT group (*n* = 12)	p value
Median stool frequency/24 h			0.949
1–3 (%)	4 (22.2)	4 (33.3)	
4–5 (%)	6 (33.3)	4 (33.3)	
6–8 (%)	6 (33.3)	3 (25)	
>9 (%)	2 (11.1)	1 (8.3)	
Urgency (<15 min) (%)	4 (22.2)	2 (16.7)	1
Anti-diarrhea medication (%)	1 (5.6)	1 (8.3)	1
Nocturnal soiling (%)	4 (22.2)	3 (25)	1
Daytime soiling (%)	2 (11.1)	1 (8.3)	1
Mean Wexner incontinence score	7.4 ± 2.7	6.8 ± 2.7	0.581
Wexner incontinence score grade			1
≤10 (%)	14 (77.8)	10 (83.3)	
>10 (%)	4 (22.2)	2 (16.7)	
Kirwan grade (%)			0.894
Grade 1 (perfect continence)	1 (5.6)	2 (16.7)	
Grade 2 (incontinence of flatus or liquids)	8 (44.4)	5 (41.7)	
Grade 3 (occasional passage of solid stools)	6 (33.3)	3 (25)	
Grade 4 (frequent incontinence of solids)	3 (16.7)	2 (16.7)	
Grade 5 (colostomy required)	0	0	

Values are means ± standard deviations or medians with ranges in parentheses.

**Table 4:** Anal function in patients without anastomotic leakage (n = 24).

Characteristic	non-TADT group (*n* = 12)	TADT group (*n* = 12)	p value
Median stool frequency/24 h			1
1–3 (%)	4 (33.3)	4 (33.3)	
4–5 (%)	5 (41.7)	4 (33.3)	
6–8 (%)	3 (25)	3 (25)	
>9 (%)	0	1 (8.3)	
Urgency (<15 min) (%)	0	2 (16.7)	0.478
Anti-diarrhea medication (%)	0	1 (8.3)	1
Nocturnal soiling (%)	1 (8.3)	3 (25)	0.59
Daytime soiling (%)	0	1 (8.3)	1
Mean Wexner incontinence score	6.0 ± 2.0	6.8 ± 2.7	0.395
Wexner incontinence score grade			1
≤10 (%)	11 (91.7)	10 (83.3)	
>10 (%)	1 (8.3)	2 (16.7)	
Kirwan grade (%)			0.572
Grade 1 (perfect continence)	1 (8.3)	2 (16.7)	
Grade 2 (incontinence of flatus or liquids)	8 (66.7)	5 (41.7)	
Grade 3 (occasional passage of solid stools)	3 (25)	3 (25)	
Grade 4 (frequent incontinence of solids)	0	2 (16.7)	
Grade 5 (colostomy required)	0	0	

Values are means ± standard deviations or medians with ranges in parentheses

## Discussion

As an extreme anus-preserving surgery, ISR can save patients with low rectal cancer from the pain of anal resection while ensuring the oncological curative effect (17, 18). However, AL is a common complication after ISR surgery. Its occurrence not only causes great distress to patients and increases their medical expenses but also affects anal function and quality of life (19, 20). Reducing postoperative AL is an important issue in the management of ISR complications. In this study, we proposed a novel drainage method to reduce the incidence of postoperative AL in patients undergoing ISR that requires handsewn anastomosis, and retrospectively analyzed its safety and efficacy.

Several factors, such as age, BMI, levels of albumin, reportedly affect the occurrence of AL after ISR (20–22). In our study, the TADT and non-TADT groups demonstrated no significant differences in their baseline characteristics (sex; age; BMI; tumor distance from the anal verge; tumor diameter; history of neoadjuvant therapy; TMN staging; levels of albumin, hemoglobin, carcinoembryonic antigen, and carbohydrate antigen 19-9; presence of hypertension or diabetes; smoking history; history of blood transfusion; and ASA score), indicating that baseline characteristics were comparable between the two groups. We analyzed the incidence of AL between the groups and found that it was significantly lower in the TADT group than in the non-TADT group. This might be related to reduced effusion surrounding the anastomosis achieved through drainage, which in turn reduces the risk of infections and provides a good healing environment.

Blood supply, tension, and local healing environment are important factors determining the quality of anastomotic healing (23–25). After ISR surgery, the reconstructed intestinal canal below the levator ani hiatus lacks an internal sphincter-like structure and is weakly attached to the surrounding tissue. At the same time, the site of anastomosis is low, and a relatively wide gap exists between the intestinal canal and external sphincter. As a result, exudates can easily collect around the anastomosis, thereby increasing the risk of infections around the area. In addition, Handsewn anastomosis stretches the anal sphincters further than stapler anastomosis, which may further increase the incidence of anastomotic complications (26, 27). Hence, indwelling drains are essential to monitor the anastomotic site and ensure good healing (28). However, in the case of ultra-low anastomosis post-ISR surgery, it may be difficult to achieve good and unobstructed drainage with conventional pelvic drainage catheters, since they have a long drainage distance, and may be potentially shifted or blocked. The GRECCAR-5 trial (13) prospectively explored the effects of conventional pelvic drainage after rectal cancer surgery. The results confirmed that the conventional drainage method did not reduce the incidence of AL and rate of secondary surgery in patients undergoing anterior resection of low rectal cancer compared with no drainage (13). Therefore, we aimed to improve the drainage method in this study. During handsewn anastomosis of the intestinal canal, two to four drainage tubes (feeding tubes approximately 10 cm long and 4 mm in diameter with multiple small lateral holes) were placed in the gap between the sutures, extending from the perianal region to the anastomotic region, through the anastomosis. Trans-anastomotic tube placement can effectively reduce effusion around the anastomosis. Using multiple drainage tubes, local effusion can be avoided with multi-directional drainage. The status of the drained fluid surrounding the anastomosis can also be visualized at the same time. Furthermore, the area can also be flushed through the drainage tubes, thereby facilitating the prevention and treatment of infections.

Drainage tubes are generally placed for about 5–7 days. The tubes can be removed if the drainage fluid is minimal, non-turbid, and no effusion or infection around the anastomosis is detected by imaging. Due to the small diameter of the drainage tubes, the gaps in the anastomosis close about two days after the tubes are removed. Further, the TADT group had a shorter hospital stay than the non-TADT group, suggesting that placement of the drainage tubes did not significantly prolong the anastomotic healing time. Moreover, the postoperative pain scores of both groups were similar, and the length of the indwelling tube outside the anus was only a few centimeters, which only minimally affected the patients’ posture and early ambulation.

Whether a protective stoma can prevent the occurrence of postoperative AL in low rectal cancer is a highly debated topic (29–31). Nevertheless, ileostomy has been found to reduce the severity of complications to a certain extent and the rate of secondary surgery associated with complications (29). Handsewn anastomosis is one of the risk factors for AL (26). Therefore, we performed terminal ileostomy on all patients in this study.

Trans-anastomotic tube placement is an invasive perianal procedure. We further explored the relationship between tube placement and long-term anal function. As reconstructed anal function tends to improve gradually over time after surgery, we evaluated the anal function of patients who had also undergone anastomosis reduction surgery, one year after rectal cancer surgery. In total, 88.2% of the patients completed the postoperative anal function evaluation. The baseline characteristics of patients were comparable, and there were no significant differences between the two groups in the Wexner incontinence score, Kirwan score, or daily defecation frequency. We further investigated the real effect of TADT on anal function by comparing TADT and non-TADT patients without AL; the difference between these two groups was not statistically significant. This indicates that trans-anastomotic tube placement may not reduce anal function in patients. However, as shown in **Table 3**, though no statistically significant difference was observed, the TADT group outperformed the non-TADT group in anal function (e.g., daily defecation frequency, urgency, and Wexner incontinence score). This is easy to explain, as the anal function in patients with AL in the non-TADT group was inferior to that in patients in the same group without AL, which is consistent with findings of previous studies (32, 33). Thus, tube placement can significantly reduce the incidence of AL, and prevents postoperative anal insufficiency caused by anastomotic complications.

There are some limitations to this study. First, since this was a single-center retrospective study, selection bias was unavoidable. Second, the small sample size makes it prone to the risk of type II error. Third, because no AL occurred in the tube placement group, we were unable to perform adjusted regression on the baseline data between the groups. Nevertheless, we put forward a novel, safe, and effective drainage approach that can significantly reduce the incidence of AL after ISR manual bowel reconstruction.

## Conclusion

As an innovative and alternative method of drainage after ISR with handsewn anastomosis, our preliminary study demonstrates that trans-anastomotic drainage may reduce the incidence of postoperative AL without adversely affecting anal function in patients. However, high-quality multicenter randomized controlled trials with large sample sizes are needed to further evaluate the safety and efficacy of TADT in preventing AL after ISR requiring handsewn anastomosis.

## Data Availability Statement

The raw data supporting the conclusions of this article will be made available by the authors, without undue reservation.

## Ethics Statement

The studies involving human participants were reviewed and approved by The Ethics Committee of Zhongnan Hospital of Wuhan University. The patients/participants provided their written informed consent to participate in this study.

## Author Contributions

Concept and design, CJ and XR. Data collection, All authors. Data analysis, XR, XZ, XX, HH. Statistical support, XR, XZ and XX. Drafting of the manuscript, XR, XZ and XX. Critical revision of the manuscript, QQ, CJ, XR, XZ and XX. All authors have read and agreed to the published version of the manuscript.

## Funding

This work was supported by grants from the Medical Science and Technology Innovation Platform of Health Commission of Hubei Province/Zhongnan Hospital of Wuhan University (grant number PTXM2019011 to CJ), the Clinical Research Special Fund of Wu Jieping Medical Foundation (grant number 320.6750.2021-11-8 to CJ), the Enginerring construction project of improving diagnosis and treatment ability of difficult diseases(oncology)(grant number ZLYNXM202012 to QQ), and Zhongnan Hospital of Wuhan University/Hubei Health Commission Joint Fund Project (grant number znpy2019086 to CJ).

## Conflict of Interest

The authors declare that the research was conducted in the absence of any commercial or financial relationships that could be construed as a potential conflict of interest.

## Publisher’s Note

All claims expressed in this article are solely those of the authors and do not necessarily represent those of their affiliated organizations, or those of the publisher, the editors and the reviewers. Any product that may be evaluated in this article, or claim that may be made by its manufacturer, is not guaranteed or endorsed by the publisher.

## References

[1] Schiessel R Karnerhanusch J Herbst F Teleky B Wunderlich M. Intersphincteric Resection for Low Rectal Tumors. Br J Surg (1994) 81(9):1376–8. doi: 10.1002/bjs.1800810944 7953423

[2] Tsukamoto S Miyake M Shida D Ochiai H Yamada K Kanemitsu Y. Intersphincteric Resection Has Similar Long-Term Oncologic Outcomes Compared With Abdominoperineal Resection for Low Rectal Cancer Without Preoperative Therapy: Results of Propensity Score Analyses. Dis Colon Rectum (2018) 61(9):1035–42. doi: 10.1097/DCR.0000000000001155 30086052

[3] Rink AD Kienle P Aigner F Ulrich A. How to Reduce Anastomotic Leakage in Colorectal Surgery-Report From German Expert Meeting. Langenbecks Arch Surg (2020) 405(2):223–32. doi: 10.1007/s00423-020-018645 32189067

[4] Park IJ Kim JC. Intersphincteric Resection for Patients With Low-Lying Rectal Cancer: Oncological and Functional Outcomes. Ann Coloproctol (2018) 34:167–74. doi: 10.3393/ac.2018.08.02 PMC6140365 30208679

[5] Jin CA Chan W Jlla B Yong S Ijp A Jrk A. Complete Intersphincteric Longitudinal Muscle Excision May Be Key to Reducing Local Recurrence During Intersphincteric Resection. Eur J Surg Oncol (2021) 47:1629–36. doi: 10.1016/j.ejso.2020.12.017 33642088

[6] Kim JC Lee JL Bong JW Seo JH Kim CW Park SH. Oncological and Anorectal Functional Outcomes of Robot-Assisted Intersphincteric Resection in Lower Rectal Cancer, Particularly the Extent of Sphincter Resection and Sphincter Saving. Surg Endosc (2020) 34(5):2082–94. doi: 10.1007/s00464-019-06989-3 31332563

[7] Martin ST Heneghan HM Winter DC. Systematic Review of Outcomes After Intersphincteric Resection for Low Rectal Cancer. Br J Surg (2012) 99(5):603–12. doi: 10.1002/bjs.8677 22246846

[8] Wang FG Yan WM Yan M Song MM. Comparison of Anastomotic Leakage Rate and Reoperation Rate Between Transanal Tube Placement and Defunctioning Stoma After Anterior Resection: A Network Meta-Analysis of Clinical Data. Ejso (2019) 45(8):1301–9. doi: 10.1016/j.ejso.2019.01.182 30738589

[9] Challine A Cazelles A Frontali A Maggiori L Panis Y. Does a Transanal Drainage Tube Reduce Anastomotic Leakage? A Matched Cohort Study in 144 Patients Undergoing Laparoscopic Sphincter-Saving Surgery for Rectal Cancer. Tech Coloproctol (2020) 24(10):1047–53. doi: 10.1007/s10151-020-02265-y 32583145

[10] Zhao S Zhang L Gao F Wu M Zheng J Bai L. Transanal Drainage Tube Use for Preventing Anastomotic Leakage After Laparoscopic Low Anterior Resection in Patients With Rectal Cancer: A Randomized Clinical Trial. JAMA Surg (2021) 156(12):1151–8. doi: 10.1001/jamasurg.2021.4568 PMC8495603 34613330

[11] Emile SH Abd El-Hamed TM. Routine Drainage of Colorectal Anastomoses: An Evidence-Based Review of the Current Literature. Gastroenterol Res Pract (2017) 2017:6253898. doi: 10.1155/2017/6253898 29158731 PMC5660819

[12] Nikolian VC Kamdar NS Regenbogen SE Morris AM Byrn JC Suwanabol PA. Anastomotic Leak After Colorectal Resection: A Population-Based Study of Risk Factors and Hospital Variation. Surgery (2017) 161(6):1619–27. doi: 10.1016/j.surg.2016.12.033 PMC5433895 28238345

[13] Denost Q Rouanet P Faucheron JL Panis Y Meunier B Cotte E. To Drain or Not to Drain Infraperitoneal Anastomosis After Rectal Excision for Cancer: The Greccar 5 Randomized Trial. Ann Surg (2017) 265:474–80. doi: 10.1097/SLA.0000000000001991 27631776

[14] Rullier E Denost Q Vendrely V Rullier A Laurent C. Low Rectal Cancer: Classification and Standardization of Surgery. Dis Colon Rectum (2013) 56(5):560–7. doi: 10.1097/DCR.0b013e31827c4a8c 23575394

[15] Denost Q Laurent C Capdepont M Zerbib F Rullier E. Risk Factors for Fecal Incontinence After Intersphincteric Resection for Rectal Cancer. Dis Colon Rectum (2011) 54(8):963–8. doi: 10.1097/DCR.0b013e31821d3677 21730784

[16] Rahbari NN Weitz J Hohenberger W Heald RJ Moran B Ulrich A. Definition and Grading of Anastomotic Leakage Following Anterior Resection of the Rectum: A Proposal by the International Study Group of Rectal Cancer. Surgery (2010) 147(3):339–51. doi: 10.1016/j.surg.2009.10.012 20004450

[17] Denost Q Moreau JB Vendrely V Celerier B Rullier A Assenat V. Intersphincteric Resection for Low Rectal Cancer: The Risk Is Functional Rather Than Oncological. A 25-Year Experience From Bordeaux. Colorectal Dis (2020) 22(11):1603–13. doi: 10.1111/codi.15258 32649005

[18] Shirouzu K Murakami N Akagi Y. Intersphincteric Resection for Very Low Rectal Cancer: A Review of the Updated Literature. Ann Gastroenterol Surg (2017) 1(1):24–32. doi: 10.1002/ags3.12003 29863144 PMC5881339

[19] Ashraf SQ Burns EM Jani A Altman S Young JD Cunningham C. The Economic Impact of Anastomotic Leakage After Anterior Resections in English Nhs Hospitals: Are We Adequately Remunerating Them? Colorectal Dis Off J Assoc Coloproctol Great Britain Ireland (2013) 15(4):E190–E8. doi: 10.1111/codi.12125 23331871

[20] Yang J Chen Q Jindou L Cheng Y. The Influence of Anastomotic Leakage for Rectal Cancer Oncologic Outcome: A Systematic Review and Meta-Analysis. J Surg Oncol (2020) 121(8):1283–97. doi: 10.1002/jso.25921 32243581

[21] Sciuto A Merola G De Palma GD Sodo M Pirozzi F Bracale UM. Predictive Factors for Anastomotic Leakage After Laparoscopic Colorectal Surgery. World J Gastroenterol (2018) 24:2247–60. doi: 10.3748/wjg.v24.i21.2247 PMC5989239 29881234

[22] Parthasarathy M Greensmith M Bowers D Wassink TG. Risk Factors for Anastomotic Leakage After Colorectal Resection: A Retrospective Analysis of 17518 Patients. Colorectal Dis (2017) 19(3):288–98. doi: 10.1111/codi.13476 27474844

[23] Armstrong G Croft J Corrigan N Brown JM Goh V Quirke P. Intact: Intra-Operative Fluorescence Angiography to Prevent Anastomotic Leak in Rectal Cancer Surgery: A Randomized Controlled Trial. Colorectal Dis (2018) 20(8):O226–O34. doi: 10.1111/codi.14257 PMC6099475 29751360

[24] Fang AH Chao W Ecker M. Review of Colonic Anastomotic Leakage and Prevention Methods. J Clin Med (2020) 9(12):4061. doi: 10.3390/jcm9124061 PMC7765607 33339209

[25] Kitaguchi D Nishizawa Y Sasaki T Tsukada Y Ikeda K Ito M. Recurrence of Rectal Anastomotic Leakage Following Stoma Closure: Assessment of Risk Factors. Colorectal Dis (2019) 21(11):1304–11. doi: 10.1111/codi.14728 31199545

[26] Cong JC Chen CS Ma MX Xia ZX Liu DS Zhang FY. Laparoscopic Intersphincteric Resection for Low Rectal Cancer: Comparison of Stapled and Manual Coloanal Anastomosis. Colorectal Dis (2014) 16(5):353–8. doi: 10.1111/codi.12573 24460588

[27] Kinugasa T Nagasu S Murotani K Mizobe T Ochi T Isobe T. Analysis of Risk Factors for Anastomotic Leakage After Lower Rectal Cancer Resection, Including Drain Type: A Retrospective Single-Center Study. BMC Gastroenterol (2020) 20(1):315. doi: 10.1186/s12876-020-01462-1 32977772 PMC7519527

[28] Qu H Liu Y Bi DS. Clinical Risk Factors for Anastomotic Leakage After Laparoscopic Anterior Resection for Rectal Cancer: A Systematic Review and Meta-Analysis. Surg Endosc (2015) 29(12):3608–17. doi: 10.1007/s00464-015-4117-x 25743996

[29] Ahmad NZ Abbas MH Khan SU Parvaiz A. A Meta-Analysis of the Role of Diverting Ileostomy After Rectal Cancer Surgery. Int J Colorectal Dis (2021) 36(3):445–55. doi: 10.1007/s00384-020-03771-z 33064212

[30] Mrak K Uranitsch S Pedross F Heuberger A Klingler A Jagoditsch M. Diverting Ileostomy Versus No Diversion After Low Anterior Resection for Rectal Cancer: A Prospective, Randomized, Multicenter Trial. Surgery (2016) 159(4):1129–39. doi: 10.1016/j.surg.2015.11.006 26706610

[31] Shimizu H Yamaguchi S Ishii T Kondo H Hara K Takemoto K. Who Needs Diverting Ileostomy Following Laparoscopic Low Anterior Resection in Rectal Cancer Patients? Analysis of 417 Patients in a Single Institute. Surg Endosc (2020) 34(2):839–46. doi: 10.1007/s00464-019-06837-4 31111210

[32] Yokota M Ito M Nishizawa Y Kobayashi A Saito N. The Impact of Anastomotic Leakage on Anal Function Following Intersphincteric Resection. World J Surg (2017) 41(8):2168–77. doi: 10.1007/s00268-017-3960-4 28289834

[33] Hain E Manceau G Maggiori L Mongin C AlDJ P Panis Y. Bowel Dysfunction After Anastomotic Leakage in Laparoscopic Sphincter-Saving Operative Intervention for Rectal Cancer: A Case-Matched Study in 46 Patients Using the Low Anterior Resection Score. Surgery (2017) 161(4):1028–39. doi: 10.1016/j.surg.2016.09.037 27894710

