# Supplementary material for: Trans-Anastomotic Drainage Tube Placement After Hand-Sewn Anastomosis in Patients Undergoing Intersphincteric Resection for Low Rectal Cancer: An Alternative Drainage Method
Source: Front Oncol. 2022 Jul 28;12:872120. doi: 10.3389/fonc.2022.872120 (PMC9365931; doi:10.3389/fonc.2022.872120)
Supplement: Supplementary file 1 [file DataSheet_1.docx]

Supplementary Table S1. Anal functional outcomes (n= 30), including detailed baseline data

| Characteristic | non-TADT group (*n*=18) | TADT group (*n*=12) | p value |
| --- | --- | --- | --- |
| Age (year) | 61.8±9.0 | 56.4±7.4 | 0.095 |
| Gender, *n* (%) |  |  | 0.772 |
| Male | 9(50) | 7(58.3) |  |
| Female | 9(50) | 5(41.7) |  |
| ASA score, *n* (%) |  |  | 0.724 |
| I | 12(66.7) | 6(50) |  |
| II | 5(27.8) | 5(41.7) |  |
| III | 1(5.6) | 1(8.3) |  |
| Body mass index (kg/m2) | 22.0±2.3 | 21.0±3.0 | 0.296 |
| Height (cm) | 167.1±6.1 | 163.9±8.4 | 0.235 |
| Weight (kg) | 61.7±8.4 | 56.8±11.7 | 0.198 |
| Preoperative serum CEA (ng/ml) | 3.6±3.0 | 3.5±3.1 | 0.951 |
| Preoperative serum CA199 (IU/ml) | 9.8±8.5 | 6.5±4.0 | 0.223 |
| Diabetes, *n* (%) | 2(11.1) | 1(8.3) | 1 |
| Hypertension, *n* (%) | 5(27.8) | 3(25) | 1 |
| Smoking, *n* (%) | 1(5.6) | 2(16.7) | 0.548 |
| Hemoglobin level (g/L) | 134.0±15.0 | 125.8±16.0 | 0.166 |
| Albumin levels (g/L) | 41.3±3.1 | 41.1±2.9 | 0.868 |
| Distance from anal verge (cm) | 3.5±0.4 | 3.4±0.4 | 0.471 |
| Chemoradiotherapy, *n* (%)* | 7(38.8) | 5(41.7) | 1 |
| Tumor size (cm) | 3.4±0.7 | 3.1±0.5 | 0.232 |
| Mean Wexner incontinence score (preoperative) | 0.3±0.6 | 0.3±0.6 | 0.889 |
| Blood transfusion | 0 | 1(8.3) | 0.4 |
| Operative time (min) | 248±16 | 257±12 | 0.121 |
| Blood loss (ml) | 40(20–300) | 62.5(25–120) | 0.104 |
| pN stage, *n* (%) |  |  | 0.632 |
| pN_0_ | 15(83.3) | 11(91.7) |  |
| pN+ | 3(16.7) | 1(8.3) |  |
| pT stage, *n* (%) |  |  | 0.901 |
| PCR* | 1(5.6) | 0 |  |
| T1 | 1(5.6) | 0 |  |
| T2 | 8(44.4) | 7(58.3) |  |
| T3 | 8(44.4) | 5(41.7) |  |
| pTNM stage, *n* (%) |  |  | 0.927 |
| PCR* | 1(5.6) | 0 |  |
| I | 8(44.4) | 7(58.3) |  |
| II | 6(33.3) | 4(33.3) |  |
| III | 3(16.7) | 1(8.3) |  |
| Median stool frequency/24 h |  |  | 0.949 |
| 1–3 (%) | 4(22.2) | 4(33.3) |  |
| 4–5 (%) | 6(33.3) | 4(33.3) |  |
| 6–8 (%) | 6(33.3) | 3(25) |  |
| >9 (%) | 2(11.1) | 1(8.3) |  |
| Urgency (<15 min) (%) | 4(22.2) | 2(16.7) | 1 |
| Anti-diarrhea medication (%) | 1(5.6) | 1(8.3) | 1 |
| Nocturnal soiling (%) | 4(22.2) | 3(25) | 1 |
| Daytime soiling (%) | 2(11.1) | 1(8.3) | 1 |
| Mean Wexner incontinence score | 7.4±2.7 | 6.8±2.7 | 0.581 |
| Wexner incontinence score grade |  |  | 1 |
| ≤10 (%) | 14(77.8) | 10(83.3) |  |
| >10 (%) | 4(22.2) | 2(16.7) |  |
| Kirwan grade (%) |  |  | 0.894 |
| Grade 1 (perfect continence) | 1(5.6) | 2(16.7) |  |
| Grade 2 (incontinence of flatus or liquids) | 8(44.4) | 5(41.7) |  |
| Grade 3 (occasional passage of solid stools) | 6(33.3) | 3(25) |  |
| Grade 4 (frequent incontinence of solids) | 3(16.7) | 2(16.7) |  |
| Grade 5 (colostomy required) | 0 | 0 |  |
| *ASA, American Society of Anesthesiologists* | | | |
| *Values are means ± standard deviations or medians with ranges in parentheses* | | |  |
| **Chemoradiotherapy, including preoperative and postoperative* | | |  |
| ** PCR, pathological complete remission* |  |  |  |

Supplementary Table S2. Anal function in patients without anastomotic leakage (n= 24), including detailed baseline data

| Characteristic | non-TADT group (*n*=12) | TADT group (*n*=12) | p value |
| --- | --- | --- | --- |
| Age (year) | 62.4±9.2 | 56.4±7.4 | 0.093 |
| Gender, *n* (%) |  |  | 1 |
| Male | 6(50) | 7(58.3) |  |
| Female | 6(50) | 5(41.7) |  |
| ASA score, *n* (%) |  |  | 0.069 |
| I | 11(91.7) | 6(50) |  |
| II | 1(8.3) | 5(41.7) |  |
| III | 0 | 1(8.3) |  |
| Body mass index (kg/m2) | 22.0±2.4 | 21.0±3.0 | 0.362 |
| Height (cm) | 166.6±5.0 | 163.9±8.4 | 0.352 |
| Weight (kg) | 61.3±8.1 | 56.8±11.7 | 0.293 |
| Preoperative serum CEA (ng/ml) | 3.7±3.0 | 3.5±3.1 | 0.917 |
| Preoperative serum CA199 (IU/ml) | 9.9±9.5 | 6.5±4.0 | 0.262 |
| Diabetes, *n* (%) | 0 | 1(8.3) | 1 |
| Hypertension, *n* (%) | 3(25) | 3(25) | 1 |
| Smoking, *n* (%) | 0 | 2(16.7) | 0.478 |
| Hemoglobin level (g/L) | 131.9±15.3 | 125.8±16.0 | 0.351 |
| Albumin levels (g/L) | 40.4±2.7 | 41.1±2.9 | 0.553 |
| Distance from anal verge (cm) | 3.5±0.4 | 3.4±0.4 | 0.633 |
| Chemoradiotherapy, *n* (%)* | 3(25) | 5(41.7) | 0.667 |
| Tumor size (cm) | 3.3±0.8 | 3.1±0.5 | 0.547 |
| Mean Wexner incontinence score (preoperative) | 0.1±0.3 | 0.3±0.6 | 0.296 |
| Blood transfusion | 0 | 1(8.3) | 1 |
| Operative time (min) | 250±18 | 257±12 | 0.28 |
| Blood loss (ml) | 32.5(20–300) | 62.5(25–120) | 0.089 |
| pN stage, *n* (%) |  |  | 1 |
| pN_0_ | 10(83.3) | 11(91.7) |  |
| pN+ | 2(16.7) | 1(8.3) |  |
| pT stage, *n* (%) |  |  | 1 |
| PCR* | 0 | 0 |  |
| T1 | 1(8.3) | 0 |  |
| T2 | 6(50) | 7(58.3) |  |
| T3 | 5(41.7) | 5(41.7) |  |
| pTNM stage, *n* (%) |  |  | 1 |
| PCR* | 0 | 0 |  |
| I | 6(50) | 7(58.3) |  |
| II | 4(33.3) | 4(33.3) |  |
| III | 2(16.7) | 1(8.3) |  |
| Median stool frequency/24 h |  |  | 1 |
| 1–3 (%) | 4(33.3) | 4(33.3) |  |
| 4–5 (%) | 5(41.7) | 4(33.3) |  |
| 6–8 (%) | 3(25) | 3(25) |  |
| >9 (%) | 0 | 1(8.3) |  |
| Urgency (<15 min) (%) | 0 | 2(16.7) | 0.478 |
| Anti-diarrhea medication (%) | 0 | 1(8.3) | 1 |
| Nocturnal soiling (%) | 1(8.3) | 3(25) | 0.59 |
| Daytime soiling (%) | 0 | 1(8.3) | 1 |
| Mean Wexner incontinence score | 6.0±2.0 | 6.8±2.7 | 0.395 |
| Wexner incontinence score grade |  |  | 1 |
| ≤10 (%) | 11(91.7) | 10(83.3) |  |
| >10 (%) | 1(8.3) | 2(16.7) |  |
| Kirwan grade (%) |  |  | 0.572 |
| Grade 1 (perfect continence) | 1(8.3) | 2(16.7) |  |
| Grade 2 (incontinence of flatus or liquids) | 8(66.7) | 5(41.7) |  |
| Grade 3 (occasional passage of solid stools) | 3(25) | 3(25) |  |
| Grade 4 (frequent incontinence of solids) | 0 | 2(16.7) |  |
| Grade 5 (colostomy required) | 0 | 0 |  |
| *ASA, American Society of Anesthesiologists* | | | |
| *Values are means ± standard deviations or medians with ranges in parentheses* | | | |
| **Chemoradiotherapy, including preoperative and postoperative* | | |  |
| ** PCR, pathological complete remission* |  |  |  |
